# Apical dominance in saffron and the involvement of the branching enzymes CCD7 and CCD8 in the control of bud sprouting

**DOI:** 10.1186/1471-2229-14-171

**Published:** 2014-06-19

**Authors:** Angela Rubio-Moraga, Oussama Ahrazem, Rosa M Pérez-Clemente, Aurelio Gómez-Cadenas, Koichi Yoneyama, Juan Antonio López-Ráez, Rosa Victoria Molina, Lourdes Gómez-Gómez

**Affiliations:** 1 Departamento de Ciencia y Tecnología Agroforestal y Genética. Facultad de Farmacia, Instituto Botánico. Universidad de Castilla-La Mancha, Campus Universitario s/n, 02071 Albacete, Spain; 2 Fundación Parque Científico y Tecnológico de Albacete. Campus Universitario s/n, 02071 Albacete, Spain; 3 Department of Agricultural and Environmental Sciences, Universitat Jaume I, 12071 Castelló de la Plana, Spain; 4 Weed Science Center, Utsunomiya University, 350 Mine-machi, Utsunomiya 321-8505, Japan; 5 Department of Soil Microbiology and Symbiotic Systems, Estación Experimental del Zaidín-Consejo Superior de Investigaciones Científicas (EEZ-CSIC), Granada, Spain; 6 Departamento de Biología Vegetal, Universidad Politécnica de Valencia, 46071 Valencia, Spain

**Keywords:** Auxin, Buds, Carotenoid cleavage oxygenases, Corm, Saffron, Strigolactones

## Abstract

**Background:**

In saffron (*Crocus sativus*), new corms develop at the base of every shoot developed from the maternal corm, a globular underground storage stem. Since the degree of bud sprouts influences the number and size of new corms, and strigolactones (SLs) suppress growth of pre-formed axillary bud, it was considered appropriate to investigate SL involvement in physiology and molecular biology in saffron. We focused on two of the genes within the SL pathway, *CCD7* and *CCD8*, encoding carotenoid cleavage enzymes required for the production of SLs.

**Results:**

The *CsCCD7* and *CsCCD8* genes are the first ones isolated and characterized from a non-grass monocotyledonous plant. *CsCCD7* and *CsCCD8* expression showed some overlapping, although they were not identical. *CsCCD8* was highly expressed in quiescent axillary buds and decapitation dramatically reduced its expression levels, suggesting its involvement in the suppression of axillary bud outgrowth. Furthermore, in vitro experiments showed also the involvement of auxin, cytokinin and jasmonic acid on the sprouting of axillary buds from corms in which the apical bud was removed. In addition, *CsCCD8* expression, but not *CsCCD7*, was higher in the newly developed vascular tissue of axillary buds compared to the vascular tissue of the apical bud.

**Conclusions:**

We showed that production and transport of auxin in saffron corms could act synergistically with SLs to arrest the outgrowth of the axillary buds, similar to the control of above-ground shoot branching. In addition, jasmonic acid seems to play a prominent role in bud dormancy in saffron. While cytokinins from roots promote bud outgrowth. In addition the expression results of *CsCCD8* suggest that SLs could positively regulate procambial activity and the development of new vascular tissues connecting leaves with the mother corm.

## Background

*C. sativus* is an economically important monocotyledonous crop producing saffron, the world’s highest priced spice [1]. In addition, the stigmas are used all over the world to treat different diseases [2]. Over the past 5000 years, farmers have selected *C. sativus* for its stigmas characterized by the accumulation of apocarotenoids [3]. *C. sativus* is a triploid perennial sterile plant, adapted to overcome a dry dormant period in the form of an underground corm. Corms remain dormant from the beginning of the dry season (April-May), when the leaves senesce and wither, to the beginning of summer (July), characterized by the formation of leaf primordia [4]. Shortly afterwards, flower morphogenesis takes place and all the flower is already differentiated by the end of August [4]. With the onset of sprouting at the end of October, the corm turns into a source organ supporting growth of the developing corm. The importance of adequate corm production is self-evident in the sterile taxon saffron, which has been reproduced vegetatively for millennia by annually replacing corms. Since almost every sprouting bud produces a corm, factors affecting sprouting are highly important for corm and flower production. Only one to three corms per mother corm are produced in one growing season through conventional methods [4]. It would take 9–10 years to produce corms required to sow one hectare from an initial corm [5]. Hence, low multiplication rates and fungal infestation of corms reduce the productivity and quality, thereby restraining the availability of planting material. A corm survives for only one season, reproducing via division into cormlets that eventually give rise to new plants, and therefore corms are indispensable for saffron propagation.

Despite its importance, the sprouting process in saffron has not been characterized precisely. As in other plants, it is thought that this process should be orchestrated by a complex interplay of phytohormone and sugar signals [6]. Abscisic acid (ABA) has been associated with the onset and maintenance of corm dormancy [7]. Gibberellins (GAs) seem to be involved in apical sprout growth after dormancy cessation, but not in dormancy maintenance [8]. So far, there is no data regarding the involvement of other hormones neither in the sprouting process nor on apical dominance in saffron. In addition, it is not known whether the corm behaves as the stem of other higher plants and follows the same behaviour regarding apical dominance. In higher plants not all of the axillary buds develop, and each bud is subjected to a decision to continue growth or to become dormant [9]. Plant hormones are major players in the control of axillary bud outgrowth. It has been known for a long time that two hormones in particular, auxin and cytokinin, are involved in this control. Auxin, which is supplied from the apical bud, indirectly suppresses axillary bud outgrowth, while cytokinins directly induce branching [10]. During the past two decades, genetic and physiological analyses in pea, *Arabidopsis* rice, and *Petunia* have predicted the involvement of an additional, novel hormone in the control of shoot branching: the SLs that act as second messengers to inhibit axillary bud outgrowth [11]. The interactions between auxin and SLs in regulation of lateral branching are complex. SLs may act by dampening auxin transport [12-14], act downstream of auxin [15], or be independent from the status of stem auxin [16] to regulate lateral branching.

SLs are long known for their role as germination stimulants for root parasitic plants [17] and pre-symbiotic branching factors for arbuscular mycorrhizal fungi [18]. In flowering plants, SLs have also been implicated in development [19] as new hormonal players in the suppression of the outgrowth of preformed axillary buds [20,21], in root system architecture, adventitious rooting, secondary growth and reproductive development [22-25]. They have been also associated with plant responses to both abiotic and biotic stresses [26-30]. However, it is on their effects on shoot branching that some of the biosynthesis and responsive genes in the SL pathway were first identified [31,32].

Several lines of evidence demonstrate that SLs are derived from apocarotenoids, and a putative biosynthetic pathway has been proposed [33]. Two carotenoid cleavage dioxygenases, CCD7 and CCD8, are involved in SL biosynthesis. CCD7 catalyses the 9,10 cleavage of 9-cis-β-carotene to produce 10′-apo-β-carotenal and β-ionone. Then, the 10′-apo-β-carotenal is cleaved by CCD8 to produce C18-ketone β-apo-13-carotenone. This compound is immediately converted by CCD8 to carlactone, through a series of different reactions [33]. Carlactone presumably serves as substrate for MAX1 enzymes which catalyze the production of the different forms of SLs found in nature [34,35].

**Figure 1:**
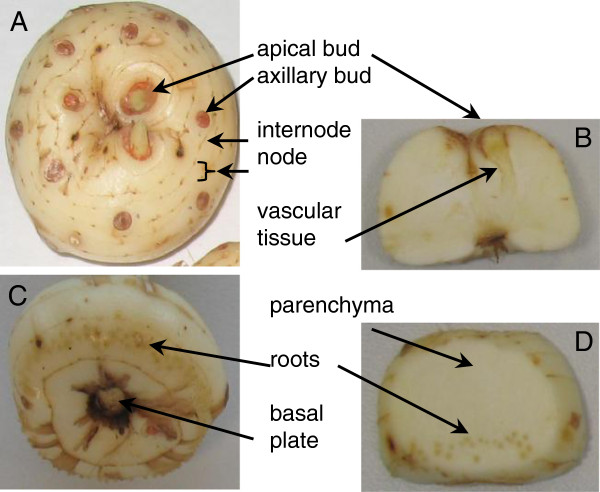
**The corm of saffron and the different parts used in this study. (A)** Upper view of the corm showing the apical meristem and the axillary buds located in the different nodes. **(B)** Transversal section of the corm through the apical meristem showing the vascular tissue and the parenchyma of the corm. **(C)** Bottom view of the corm showing the basal plate which connected the corm to the mother corm, along with the roots. **(D)** Transversal section of the corm showing the roots and the parenchyma tissue.

Orthologues of *CCD7* and *CCD8* have been characterized in plants such as Arabidopsis, pea, petunia, rice, chrysanthemum, kiwi, and tomato [14,25,32,36-40]. In addition, a *CCD8* orthologue has been isolated from the moss *Physcomitrella patens*[41].The saffron corm is a stem-derived organ formed by shortened nodes and internodes. Mature saffron corms usually show one to three apical dominant buds which will sprout in the following season plus many axillary dormant buds (Figure 1). Each axillary bud has the same developmental potential as the primary shoot apical meristem in that it can produce a growing shoot axis. However, axillary buds enter a dormant state after forming only a few leaves. The large number of axillary buds in the corm system allows the plant to recover quickly from damage and to adjust its growth according to environmental inputs. Apical dominance acts as a plant survival mechanism by providing a reservoir of meristems that can replace the damaged primary shoot. This mechanism works when the primary shoot is damaged or removed through disease, herbivore grazing, or pruning. In some plants, apical dominance can also be released depending on developmental programs. Dormant axillary buds start their outgrowth after the primary bud differentiates into the determinate organ, such as a flower. These additional shoots are important for increasing the total number of leaves or flowers to be more fruitful. However, this is not the case of saffron, where no activation of additional axillary buds is induced.

In view of the importance of sprouting in saffron and the potential impact of apical dominance on the number of corms produced, it was considered timely to investigate the role of SLs in this process, including the control of axillary branching, which can be extended to other plants propagated by corms. In this study we have identified two saffron genes required for SL biosynthesis as well as the involvement of key growth regulators in this process.

## Results

### Characterization of axillary bud sprouting in saffron

**Figure 2:**
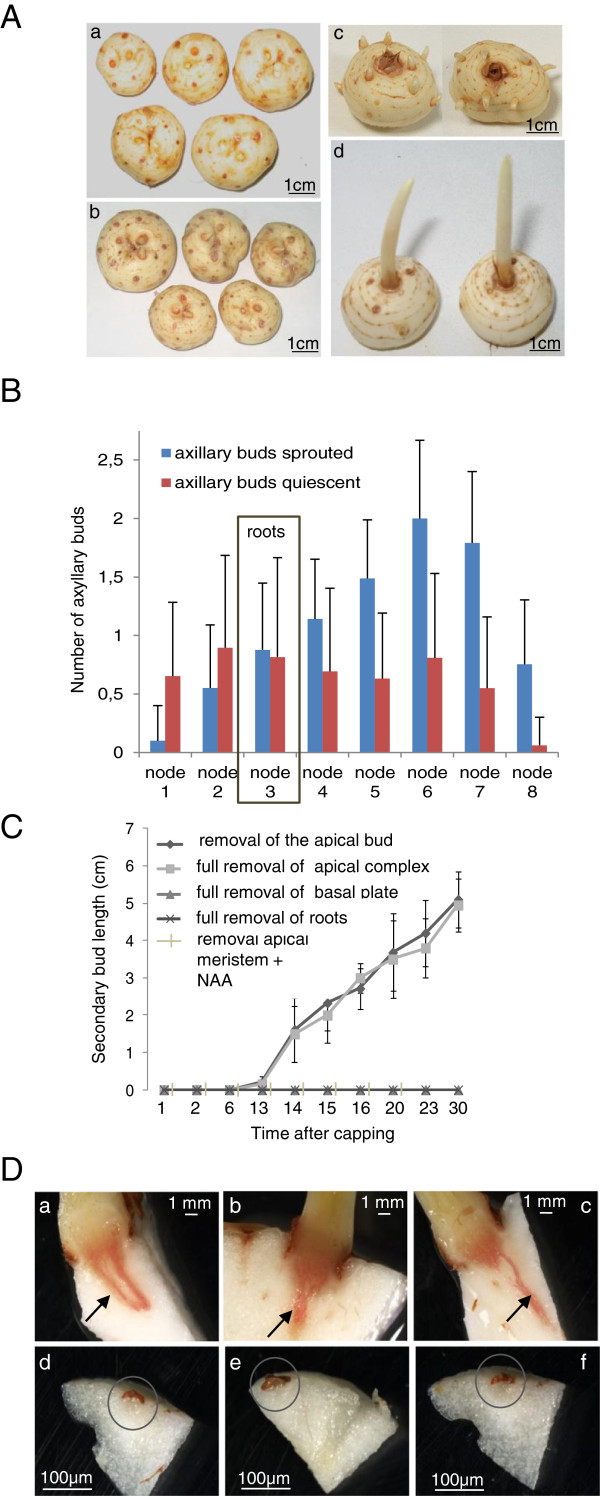
**Dominance of the apical meristem in nondormant *Crocus sativus *corms. (A)** removal of the apical bud allowed the sprouting of the axillary buds. **a)** corms with the apical meristems removed. **b)** intact corms. **c)** corms with sprouted secondary axillary bud 13 days after apical meristem removal. **d)** intact corms, as control, 13 days after the initiation of the decapitation experiment. **(B)** number of axillary buds sprouted or quiescent in each node after decapitation of the apical meristem. In each treatment, 25 corms were decapitated. Error bars represent SD of 25 replicates. **(C)** removal of the apical bud with or without other corm parts and results in sprouting of axillary buds. In each treatment, 25 corms were decapitated. Error bars represent SD of 25 replicates. **(D)** vascularization in the sprouted axillary buds of saffron corms **(a-c)**. Absence of vasculature in quiescent axillary buds **(d-f)**. Buds were sectioned by hand and sections were stained for lignin with phloroglucinol-HCl. Lignin staining is red. Pictures were taken under a dissection microscope.

Axillary buds are usually dormant, and are inhibited by auxin produced by the apical meristem. We checked whether the saffron corm exhibited classical stem-like behaviour and also investigated the role of bud apical dominance in determining lateral bud dormancy release and sprouting. Decapitation of the apical bud of the corm induced a loss of apical dominance and after 15 days, all axillary buds were sprouted (Figure 2A). We determined the number of axillary buds sprouted in each node and the number of axillary buds that remained quiescent 35 days after decapitation of the main bud (Figure 2B). The first node was set as the one that was occupied by the basal plate. The total number of axillary buds per node was increased from the bottom to the apical meristem, reaching the maximum at node 6 (Figure 2B). In the first and second nodes the percentage of sprouted buds was 13.5 and 38%, respectively. The roots were formed in the third node in 98% of the analysed corms, and in this node the percentage of sprouted axillary buds was 51.8%. From this node onward, the pattern of sprouting changes, with an increasing number of sprouted buds in comparison with the buds that remain quiescent in each node, from 62% in the fourth node up to 92.7% in the eighth node (Figure 2B). Practically all these sprouted axillary buds will form a new replacement corm, resulting in an increase in the number of corms (Additional file 1: Figure S1). To characterize this process in depth, the corms were subjected to the following treatments: (i) decapitation of the apical bud; (ii) full removal of the apical complex; (iii) full removal of secondary quiescent buds; (iv) full removal of the basal complex with or without apical bud removal; and (v) full removal of the adventitious roots with or without apical bud removal. The sprouting was followed for 30 days. Decapitation of the apical bud induced accelerated loss of apical dominance, affecting axillary bud growth. After 15 days, all axillary buds were sprouting (Figure 2C). The same pattern was observed when the apical complex (the apical bud plus underlying tissues) was removed. As expected, when auxin was applied to the decapitated apex, axillary bud sprouting was suppressed (Figure 2C and Additional file 2: Figure S2). Interestingly, the removal of the basal plate inhibited the growth of the apical and axillary buds. The same results were obtained when the adventitious roots were removed (Figure 2C). However, in this case, when corms were transferred to a humid surface allowing the regeneration of the roots, the growth of the apical bud was restarted and secondary buds sprouted in the corms when the apical bud was removed. These experiments emphasize the importance of apical bud presence and viability in the control of axillary bud growth but also the involvement of the roots on bud sprouting. Roots have been considered to be the source of cytokinins, and cytokinins induced the fast growth of axillaries buds in decapitated corms (Additional file 2: Figure S2).

Several works have pointed out that after decapitation, the axillary bud needed to form a vascular connection before it could grow. We investigated whether a vascular connection is already present in the axillary buds of saffron. Axillary buds in saffron are covered by a few leaves, thus these axillary buds might initiate a few leaves and then become developmentally arrested or dormant because the terminal bud inhibits their growth. These dormant axillary buds need to develop a vascular connection previously to be able to grow, and this process is relatively slow (Figure 2D) in comparison with other systems studied [32]. Quiescent axillary buds in saffron have not developed the vascular connections with mother corm (Figure 2D), but once the sprouting of these axillary buds is induced, the development of that vasculature begins (Figure 2D).

### Hormones in saffron buds

**Figure 3:**
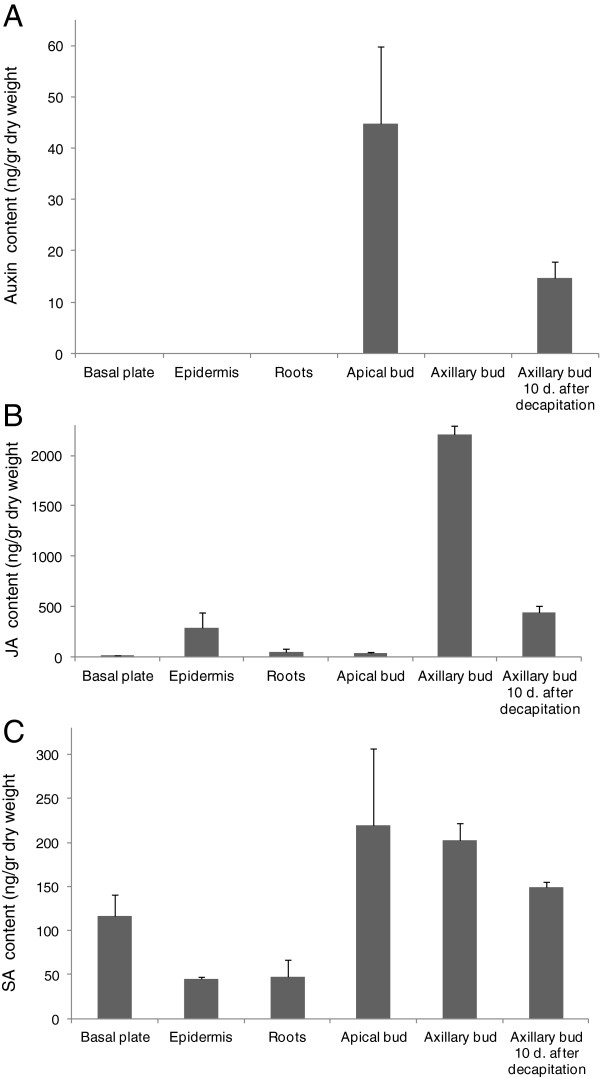
**Hormonal contents in different parts of saffron corms.** Each column represents the mean ± of two to four replicates of independently harvested plant material. **(A)** Auxin content in the different samples. **(B)** Jasmonic acid (JA) content in the different samples. **(C)** Salicylic acid (SA) content in the analyzed samples.

Apical dominance was one of the first developmental phenomenon shown to be regulated by plant hormones. Auxin, derived from the apical bud, moves basipetally in the stem through the polar auxin transport stream and inhibits the growth of axillary buds, whereas cytokinin derived mainly from the roots, promotes the outgrowth [42,43]. In order to characterize the hormonal regulation of axillary bud sprouting in saffron corms, the level of auxin and several other hormones were measured in the different parts of the corm (Figure 3). High levels of auxins were detected in the apical buds, while auxins were not detected in the other tissues tested, including axillary buds (Figure 3A). Interestingly, a significant increase in auxin content was detected in axillary buds after removal of the apical meristem (Figure 3A), suggesting a reorganization of the dominance after decapitation. By contrast, the highest level of jasmonic acid (JA) was detected in the quiescent axillary buds, decreasing ten days after decapitation of the apical bud (Figure 3B). The highest level of salycilic acid (SA) was detected in apical and axillary buds followed by the basal plate (Figure 3C).

### SLs in saffron corms

**Figure 4:**
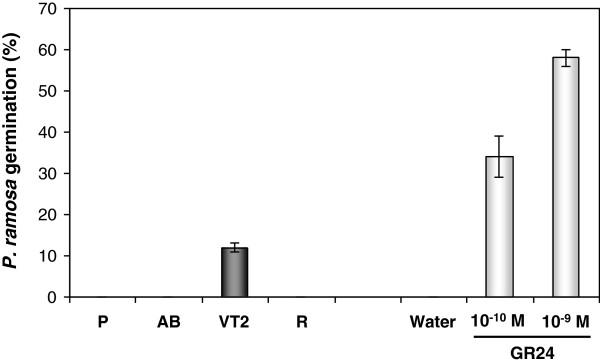
**Analysis of root-extractable strigolactones quantified by a germination bioassay in different in different corm tissues.** Germination of *P. ramosa* seeds induced by extracts from: P, parenchyma; AB, axillary bud; VT2, vascular tissue from axillary buds and R, roots. GR24 (10–9 and 10–10 M) and demineralized water were used as positive and negative controls, respectively. Bars represent the means of two pools from five independent samples (±SE).

To assess the presence of SLs in saffron, several parts were dissected and tested for the induction of germination of *Phelipanche ramosa* seeds (Figure 4). Apical buds, axillary latent buds and sprouted axillary buds, external cover (the external surface of the corms without buds), roots, basal plate, and vascular tissue from apical buds, decapitated buds and axillary bud extracts were applied to *P. ramosa* seeds, and germination was scored after 7 d. The extracts of the main vascular tissue from the apical buds induced 11.5% germination, whereas germination was not induced using extracts from the other tissues. The synthetic SL GR24 (10^-9^ to 10^-10^ M) was used as a positive control and induced 58% and 34% germination, respectively, whereas water (negative control) induced no germination (Figure 4).

### Identification of the first saffron CCD7 and CCD8 genes

**Figure 5:**
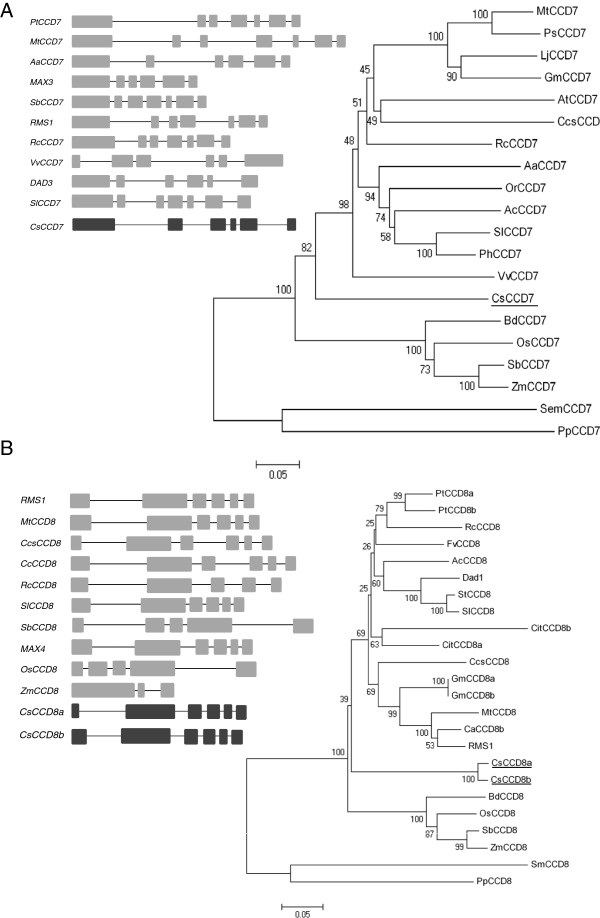
**Gene structures of *CsCCD7 *and *CsCCD8 *and the phylogenetic relationship with CsCCD7 and CsCCD8 homologues from other plant species. (A)** The postulated intron/exon structure for *CsCCD7* and positions of the introns in orthologues of *CsCCD7* is shown in the left side of the figure. In the right side a representative phylogenetic tree of CCD7 proteins from different plant species is shown. **(B)** The postulated intron/exon structure for *CsCCD8* and positions of the introns in orthologues of *CsCCD8* is shown at the left side. In the right side a representative phylogenetic tree of CCD8 proteins from different plant species is shown. Exons and introns are shown as boxes and lines, respectively. The present trees were obtained after alignment of full-length CCDs sequences using ClustalW and clustering with the neighbour-joining method. Accession numbers are as follow: SlCCD7 (ACY39882.1), PhCCD7 (ACY01408.1), AcCCD7 (ADP37985.1), ZmCCD7 (NP_001183928.1), RcCCD7 (XP_002511629.1), VvCCD7 (XP_002274198.1), AaCCD7 (ADB64459.1), PsCCD7 (ABD67496.2), GmCCD7 (ADK26570.1), AtCCD7 (NP_182026.4), CcsCCD7 (ADM18968.1), SmCCD7 (XP_002984696.1), OsCCD7 (EAY95081.1), LjCCD7 (ADM88552.1), MtCCD7 (XP_003622555.1), BdCCD7 (XP_003581501.1), PpCCD7 (ADK36680.1), PtCCD8a (XP002309543), PtCCD8b (XP002324797), AcCCD8 (GU206812.1), AtCCD8 (AT4G32810), BdCCD8 (LOC100831734), MtCCD8 (Medtr3 g127920), OsCCD8 (Os01 g0746400), Dad1 (AY743219), RMS1 (AY557342), SbCCD8 (Sb03 g034400), ZmCCD8 (GRMZM2G446858), RcCCD8, SlCCD8 (NP_001266276.1), StCCD8 (XP_006359761.1), CitCCD8a (KD079823), CitCCD8b (XP_006476130, CaCCD8b (XP_004501157), PpCCD8 (ADK36681.1), SmCCD8 (XP_002972693.1), GmCCD8b (XP_003522713), Gm CCD8a (XP_003522713.2). Branch support is under 5000 Bootstrap replicas.

SLs are a new class of plant hormones that have been shown to be involved in the regulation of the outgrowth of preformed axillary buds. In order to study the relationship between apical dominance and SLs in saffron, the *CCD7* and *CCD8* genes were isolated using a combination of degenerate primer PCR and gene walking. Saffron *CCD7*, hereafter designated as *CsCCD7*, contains five introns (Figure 5A), and the 1912 bp of the coding sequence encodes a protein of 591 amino acids with a predicted pI of 6.6 and 66.17 kDa (GenBank accession number KJ361477). The ChloroP 1.1 program predicted a 50-amino acid, N-terminal transit peptide, consistent with plastid localization of CCD7 [44]. CsCCD7 showed the highest homology with *Solanum lycopersicum* CCD7 protein (67% identical). In the case of the *CCD8*, two .1pt?>different genes were isolated from saffron, designated as *CsCCD8a* and *CsCCD8b*, which differ in the sequence of the first exon and intron. *CsCCD8a* was predicted to have six exons (Figure 5B), 3151 nucleotides and a coding sequence of 1533 nucleotides encoding a protein of 511 amino acids with a predicted pI of 6.6 and 57.17 kDa (GenBank accession number KJ361478). *CsCCD8b* was predicted to also have six exons, 3195 nucleotides and a coding sequence of 1671 nucleotides encoding a protein of 557 amino acids with a predicted pI of 6.1 and 62.03 kDa (GenBank accession number KJ361479). Both CsCCD8 proteins showed 83% identity with DAD1 (CCD8 from *Petunia hybrida*) and 76% to D10 (CCD8 from *Oryza sativa*), and were predicted to be localized in plastids using the ChloroP 1.1 program, consistent with plastid localization of CCD8 [45].

As part of the characterization of CsCCD7, CsCCD8a and CsCCD8b, amino acid sequence alignments were carried out, in order to build a phylogenetic tree using the CCD7 and CCD8 protein sequences from a variety of plant species (Figure 5A and B). This analysis showed that CsCCD7 was closer to the eudicot sequences than to the grass sequences (Figure 5A) while CsCCD8a and CsCCD8b were in a cluster separate from the eudicots (Figure 5B and Additional file 3: Figure S3).

### Gene expression of CsCCD7and CsCCD8

**Figure 6:**
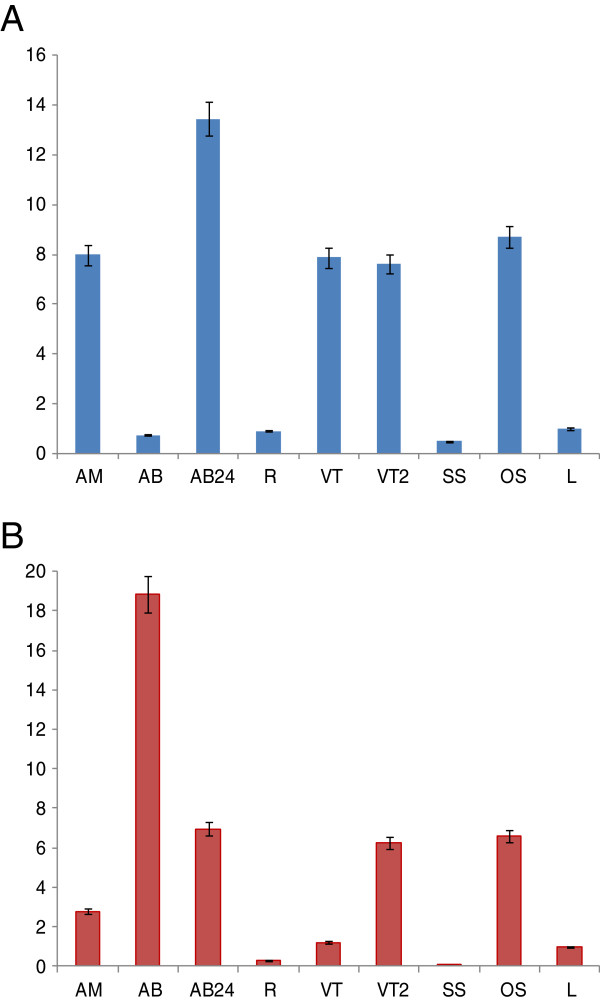
***CsCCD7 *and *CsCCD8 *transcript accumulation (normalised to the saffron household gene RSP18). (A)** relative gene expression of *CsCCD7* in different plant tissues. **(B)** relative gene expression of CsCCD8 in different plant tissues. AM, apical bud; AB, axillary bud; AB24, axillary bud 24 hr after decapitation; R, adventitious root; VT, vascular tissue from apical meristem; VT2, vascular tissue from secondary buds; SS, senescent stigma; OS, orange stigma; L, leaf. Values are means of three technical replicates ± SE, normalized to the internal control gene.

To determine where *CsCCD7* and *CsCCD8* were expressed, the pattern of *CsCCD7* and *CsCCD8* transcript abundance was determined in different tissues using quantitative real-time RT-PCR (qPCR). *CsCCD7* expression was readily detected in all the analyzed tissues, but showing different expression levels. At the level of buds, the highest expression was detected in the axillary buds 24 h after removal of the apical bud, followed by the levels of expression in the apical bud (Figure 6A). The levels in the vascular tissue from the apical buds or from the sprouted axillary buds were similar (Figure 6A). Interestingly, high expression levels were observed in the orange stigma in contrast with the low levels detected in the senescent stigma. The expression levels were also low in leaves and adventitious roots (Figure 6A). The combined levels of both *CsCCD8* transcripts in mRNA extracted from different tissues were examined using primers that do not discriminate between the two different copies/alleles. The highest levels were detected in the quiescent axillary buds (Figure 6B). However, these levels were drastically reduced in the axillary buds 24 h after removal of the apical bud. The expression levels in the apical bud were much reduced in comparison with the expression levels in the quiescent axillary buds, suggesting that the levels of *CsCCD8* are reduced during the sprouting process (Figure 6B). The expression of *CsCCD8* in the vascular tissue from the apical buds was lower than that in the newly developed vascular tissue of the sprouted axillary buds, but higher than that observed in adventitious roots (Figure 6B). Similarly to *CsCCD7*, a relatively high expression of *CsCCD8* was detected in orange stigma, while this expression was reduced in senescent stigma (Figure 6B).

## Discussion

Almost all bulbous plant species are monocots, including economically important plants such as saffron, tulip, onion, garlic and lily. Their vegetative propagation constitutes the most relevant process for agronomical improvements to markedly increase the potential number of bulblets, while the control of dormancy is crucial to solve many problems associated with the storage and distribution of these crops. SLs play a key role in both processes, development of new buds and sprouting inhibition by inhibition of bud outgrowth [20,21,46,47] and several genes involved in the biosynthesis and signalling of SLs have been identified from a diverse range of species [48], although excluding bulbous plants. In this paper, we describe and analyse the sprouting process in saffron induced by decapitation, as well as the involvement of SLs in this process through the isolation and characterization of two key genes in SL biosynthesis, *CsCCD7* and *CsCCD8*.

### Isolation of the saffron CCD7 and CCD8 genes

The genes so far identified that control branching are frequently conserved between species. In particular, two carotenoid cleavage dioxygenase genes, *CCD7* (*MAX3/RMS5/DAD3/D17-HTD1*) and *CCD8* (*MAX4/RMS1/DAD1/D10*), involved in SLs biosynthesis, appear to be well conserved among the plant species studied. To characterize the SL pathway in saffron, the saffron orthologues of *CCD7* and *CCD8* were isolated, and their orthology was confirmed by phylogenetic analysis. Analyses of the genomes of several plants species showed that *CCD7* is a single copy gene, which also seems to be the case in saffron, analysed in this study. However, in most of the analysed genomes *CCD8* is present as a multicopy gene [49] (Additional file 3: Figure S3). In saffron, our results suggest that there are at least two loci encoding *CCD8*.

Despite the high functional conservation of the *CCD7* and *CCD8* genes between species, there are interesting differences in the expression patterns. *CsCCD7* and *CsCCD8* were expressed in all tissues and organs examined, with very low expression levels in adventitious roots. In *Arabidopsis*[45], petunia [38], pea [50], kiwi [40] and tomato [25], root expression of *CCD8* is at least 10 times higher than that in the shoot. By contrast, in rice [51] and chrysanthemum, shoot expression exceeds root expression [14], whereas in rose no expression is detected in roots [52]. In *Arabidopsis*, *CCD7* expression was high in roots, [37] although recently, the highest expression has been detected in seeds and in the stem vascular tissue [53]. In rice *CCD7* is expressed in both shoot and root tissues being mainly expressed in vascular bundle tissues throughout the plant [54]. In petunia, its expression is higher in nodes and internodes [55] and in tomato, *CCD7* expression in green tissue is far less than that detected in the stem and in the roots [39]. These differences may reflect different contributions of the root and shoot in the SL-regulation of shoot branching in different species.

### Apical dominance in saffron in relation with the hormone content and the positional effect of axillary bud sprouting

Apical dominance is thought to result from the developmental arrest of lateral buds caused by auxin, which is basipetally transported from the shoot apical meristem [56]. This notion is supported by the fact that apical dominance is maintained if an excised apex is replaced by an exogenous source of auxin. Auxin derived from the shoot apex might control lateral bud outgrowth by the action of SLs, relaying the inhibitory signal from the main stem into the buds [15]. This process has not previously been studied in depth in saffron. As observed in other plant systems, apical dominance in saffron was released by excision of the apical bud, which was the main source of auxin among the tested organs and tissues, and conversely, apical dominance was maintained by the application of auxin to the cut surface of the decapitated corm.

In addition, the importance of roots has been shown, possibly as a source of cytokinin for sprouting of apical and secondary buds, in the latter, when apical dominance has been lost (Additional file 2: Figure S2).

The buds more closely located to the apical bud were more active, in terms of sprouting, than those distant from the apical bud, suggesting that the outgrowth potential of each axillary bud is related to its position in the corm. The decision of which buds activate first depends on the local bud competitiveness, which is probably determined by the local environment and developmental state of the bud [57]. On the other hand, a perception of altered light quality, in particular a decreased ratio of red light to far-red light (R/FR) perceived by the phytochrome B has a key role in this process, inducing an inhibition of bud outgrowth [58-60]. However, on our experimental system, considering the corms as an underground organ it is not clear whether the buds nearer the apex are exposed to some sunlight. However, this positional effect has been observed in other plants such as pea, where only one bud per axil is released at the upper nodes when branching is promoted by decapitation [61,62], and appears to be determined by a balance between several hormones [63].

We also measured the levels of JA in several tissues of saffron. JA and its derivatives have been implicated in stress-induced responses and have also been shown to inhibit plant growth and mitosis [64]. The highest levels of JA were detected in quiescent axillary buds, but such levels were reduced after the decapitation of the apical bud and were undetectable in apical buds. These data suggest that JA could play a prominent role in bud dormancy in saffron. Similarly, JA has recently been shown to be involved in bud dormancy in apricot [65] and orchids [66]. The observed pattern of JA was opposite to the one observed for auxin. Cell elongation and meristem activity required for plant growth are regulated by auxins. Interestingly, JA shows extensive crosstalk with auxin and down-regulates PIN1 and PIN2 protein levels [67], suggesting a possible role of this hormone in PIN protein trafficking and auxin transport, as suggested for SLs [48]. In agreement with this observation, it was shown that a gain in function mutation in *IAA8* induced more lateral branches and decreased shoot apical dominance by reducing JA levels [68].

Endogenous cytokinins (CKs) can enter axillary buds and promote their outgrowth by promoting the cell cycle. CKs are synthesized throughout the plant, but the origin of CKs in bud regulation is still under debate [69]. It has been shown that CKs produced in the roots are transported through the xylem [70,71] and exert their action on different tissues. Removal of roots in saffron interrupts bud outgrowth and the development of new roots restart the sprouting process. Due to the involvement of CKs in outgrowth bud promotion (Additional file 2: Figure S2), it is likely that CKs produced in the roots are responsible for this process in saffron as shown in other plant systems [72].

SA levels have been shown to change between dormant and waking saffron corms [73], suggesting its ability to break dormancy. Therefore, we determined the levels of SA in apical buds, axillary buds and in axillary buds 10 d after removal of the apical bud. However, we did not detect significant differences among the samples, suggesting that endogenous level of SA in buds is not involved in the control of paradormancy.

### SLs in saffron corms and the roles of CsCCD8 and CsCCD7in sprouting and vascular tissue formation

Another major player in the control of shoot branching are the SLs [74], originally identified as germination stimulants for root parasitic plants [75]. SLs, together with auxin, have been shown to have an inhibitory role on shoot branching [20]. The auxin transport auto-inhibition hypothesis [76] proposed that organs remain dormant because they are not able to export their own auxin into the stem polar auxin flow. Once axillary bud dormancy is broken, bud outgrowth may depend on the establishment of auxin transport from the bud via a process involving canalization, which is controlled by the strength of the polar auxin transport stream and or may require an auxin-regulated second messenger [16]. The SLs were proposed to act as regulators of auxin transport by reducing the expression and/or plasma membrane localization of auxin transporters [77,78].

In the present work, the germination stimulatory activity of *P. ramosa* seeds of different extracts was tested, and the stimulatory activity, albeit low, was only detected in the fractions from vascular tissue developed from the apical bud indicating the presence of SLs in this tissue. The absence of activity in the extracts from other tissues is most probably due to the presence of SLs at extremely low levels. Grafting studies performed in several species showed that a wild-type rootstock grafted to either a *ccd7* or *ccd8* mutant scion was able to restore wild-type branching patterns, indicating that SL was produced in the roots [31,79]. However, wild-type shoots on mutant roostocks also have near-wild-type branching patterns [31,32,79]. In addition, wild-type epicotyl interstock grafts into *rms1* and hypocotyl grafts into *Arabidopsis max3* are also able to reduce branching [80], indicating that biosynthesis of SLs is not limited to the root system. Further, the expression of *CsCCD7* and *CsCCD8* in the vascular tissues connecting sprouting buds with the mother corm, suggested that SLs are also synthesized in the stem vascular system in saffron. In addition, the SL profile found in tomato root exudates is different from that found in xylem sap [81], suggesting that different SLs could be produced in different tissues in which they have different biological functionalities.

The expression patterns of *CsCCD7* and *CsCCD8* were analysed in apical and in axillary buds at two different developmental stages, quiescent and 24 h after elimination of the main apical bud. For both genes expression in apical buds was higher than in roots or leaves. However, in axillary buds the expression patterns of both genes were clearly different. Although *CsCCD8* showed the highest levels of expression in the quiescent axillary buds, the expression levels of *CsCCD7* were very low in this tissue. In *Arabidopsis*, expression of *CCD8* has also been reported to be relatively high in nodal tissue close to the buds, while in rice, *CCD7* was mainly found at the node of the stem where the axillary meristem initiates [54]. In fact, the *CsCCD8* transcript levels in the axillary buds were rapidly down regulated by decapitation of the apical bud, although the expression of *CsCCD7* was up regulated, as has been observed in potato [82]. Previous data on *CCD7* and *CCD8* expression patterns in other plant systems revealed that decapitation results in decreased expression of these genes in the stem and in the axillary bud [14,15,32,51,83]. Even though this was the case for *CsCCD8*, *CsCCD7* showed the opposite behaviour. This result suggests that SL production related to bud dormancy is most probably controlled at *CsCCD8* level.

Moreover, bud auxin export is also a prerequisite for the formation of vascular connections to the stem vasculature in inhibited buds [16,84]. In the quiescent axillary buds of saffron the vasculature is not well developed, and the sprouting process is accompanied by the development of this system. The leaf primordia of these quiescent axillary buds are not a source of auxins. However, once the bud start to grow, buds synthesize auxin, as observed in other plants [85], and its export may enhance vascular connections and nutrient flow to further stimulate the growing bud. Interestingly, it is in the vasculature of the axillary buds where the *CsCCD8* expression levels were enhanced, compared with the vasculature of apical buds, although *CsCCD7* levels remained practically unchanged, but high. Recently [86], it has been provide evidence that SLs positively regulate cambial activity. The expression pattern of the studied genes suggests that most probably SL or carlactone production in this vascular tissue is controlled at the level of *CsCCD8* but not *CsCCD7*. In agreement with this, several reports point out to the involvement of CCD7 in the formation of other apocarotenoids [87,88]. Both CCD4 and CCD7 are currently candidates to deliver C_27_ intermediates for CCD1, which has been suggested to act preferentially over apocarotenoids [89]. Consistent with this additional role for CCD7, strongly elevated levels of *CCD7* can be found in green tomato fruits, from which SLs have not been detected [39] and in panicles of rice [51]. Interestingly, expression of *D27*, a β-carotene isomerase that converts all-*trans*-β-carotene into 9-*cis*-β-carotene, which is cleaved by CCD7, is also high in panicle, but low in roots [90].

### *Involvement of CsCCD7* and *CsCCD8 in stigma development*

Unexpectedly, *CsCCD7* and *CsCCD8* transcripts were detected at relatively high levels in the stigma tissue. The abundance of both transcripts in immature orange stigmas exceeded that seen in vascular tissues, leaves and roots. *CsCCD7* and *CsCCD8* expression in the developing stigma suggests potentially interesting novel function(s) for these enzymes and of SLs. The female organs of *C. sativus* consist of a trilocular ovary, a very long style, and 3 red stylar branches forming the stigmas folded to give a trumpet-like structure [91]. This structure is already present in the earlier developmental stages of the stigma, which is approximately 2 mm in length [4], and the cells continuously elongate until the stigma is fully developed reaching a final length of 30 mm [92]. Auxins participated in the elongation of the floral tube in *Crocus*[93] and they are probably involved in the style elongation. Concomitant with cell elongation, the development of the vasculature of the stigma takes place, and SLs could be actively participating in this process, explaining the expression of *CsCCD7* and *CsCCD8* during the development of the stigma. Once the stigma is developed, the expression of both genes and probably the production of SLs drops in the senescent stigma. A putative function for SLs in flower development was already expected as the petunia *ccd8/dad1* mutant was reported to have smaller flowers [38] while in *SlCCD8* knock-down lines sepals, petals and anthers were smaller than in wild-type plants [25], suggesting that SL deficiency affects flower development.

## Conclusions

The molecular and hormonal regulations on bud sprouting in bulbous plant species are largely unknown, but are fundamental for their propagation. We have determined that the corm behaves as the stem of other higher plants and follows the same behaviour regarding apical dominance. In this study, jasmonic acid, auxin and SLs are associated with the negative regulation of axillary bud outgrowth in saffron, while cytokinins positively regulates bud outgrowth after decapitation. Two key genes in SLs biosynthesis, *CCD7* and *CCD8,* were cloned from saffron. *CsCCD8* may play an important role in the control of apical dominance but also in the control of vascular and stigma development. As the perception and signalling mechanisms for SLs pathway are becoming understood in other plant species, more work needs to be done to understand the mechanism of regulation of the sprouting process in saffron.

## Methods

### Chemicals and plant materials

Chemicals and reagents were obtained from Sigma-Aldrich unless otherwise stated. Diverse organs and plant tissues from *C. sativus* grown under field conditions in Tarazona de La Mancha, Spain, were used throughout the experiments. Corms, stigmas and buds at different developmental stages, and leaves were collected for the experiments. All tissues were frozen in liquid nitrogen and stored at -80°C until required.

### Sprout release assay

Saffron corms of 15–20 g collected in September were used for the sprouting experiments. Apical buds and other plant tissues were excised with sterile surgical blades and the outgrowth of the axillary buds in each corm was scored daily during a period of 30 days. 1-Naphthaleneacetic acid (NAA) was used at 50 μM concentration.

### Histochemical staining of lignin

Hand-cut sections of corms were stained for lignin detection with phloroglucinol. Phloroglucinol-HCl reagent was prepared by mixing 2 volumes of 2% (w/v) phloroglucinol in 95% ethanol with 1 volume of concentrated HCl. All photographs were taken within 30 min of staining.

### Hormone levels

Saffron corms collected in September were dissected in different parts. Apical bud, secondary or axillary buds, roots, basal plate, nodes, nodes, external surface and parenchyma tissue were obtained (Figure 1), immediately frozen in liquid nitrogen and lyophilized. Hormone extraction and analysis were carried out as follows: frozen dry plant material was extracted in distilled water after spiking with 100 ng of dihydrojasmonic acid, [2H^4^]-salicylic acid. After centrifugation at 4000 × g at 4°C, supernatants were recovered and pH adjusted to 3.0 with 30% acetic acid. The acidified water extract was partitioned twice against 3 ml of di-ethyl ether. The organic layer was recovered and evaporated under vacuum in a centrifuge concentrator (Speed Vac, Jouan, Saint Herblain Cedex, France). The dry residue was then resuspended in a 10% MeOH solution by gentle sonication. The resulting solution was filtered through regenerated cellulose 0.22 μm membrane syringe filters (Albet S.A., Barcelona, Spain) and directly injected into a UPLC system (Acquity SDS, Waters Corp., Milford, MA, USA). Separations were carried out on a C18 column (Macherey-Nagel, 1.8 μm particle size, 50 × 2.1 mm, Scharlab, Barcelona, Spain) using a MeOH:H_2_O (both supplemented with 0.1% acetic acid) gradient at a flow rate of 300 μl min^-1^. Hormones were quantified with a Quattro LC triple quadrupole mass spectrometer (Micromass, Manchester, UK) connected online to the output of the column through an orthogonal Z-spray electrospray ion source.

### Germination bioassay with P. ramosa seeds

As described above, SLs are germination stimulants of root parasitic plant seeds. Because of this germinating activity, bioassays based on seed germination of root parasitic plants can be used as a reliable indirect way to quantify the levels of SLs produced by plant roots, especially in plants where they have not been characterized, as in saffron. SLs from the different saffron corm tissues were extracted as described [94]. Briefly, 0.3 g of each corm tissue were ground in a mortar with liquid nitrogen and extracted twice with 0.3 mL of 50% acetone in a 2 mL eppendorf tube. Tubes were vortexed for 2 min and centrifuged at 4ºC for 5 min at 8000 g in a table top centrifuge. The organic phase was carefully transferred to 2 mL glass vials and stored at -20°C until use. Germination bioassays with *P. ramosa* seeds (kindly provided by Dr. Mauricio Vurro, Instituto di Scienze delle Produzioni Alimentari, Bari, Italy) were preconditioned for 12 d at 21ºC. Then, aliquots of 50 μl of extracts were added to two discs bearing approximately 100 preconditioned seeds and incubated at 25°C. The synthetic germination stimulant GR24 and demineralised water were included as positive and negative controls in the bioassay. After 7 days, the germinated and non-germinated seeds were counted using a binocular microscope.

### Cloning CsCCD7 and CsCCD8

To facilitate genetic analysis of SL functioning in saffron, we focused on identifying steps in the saffron SL biosynthetic pathway. Partial coding sequences of saffron *CCD7* and *CCD8* were recovered from *C. sativus* gDNA using degenerate primers (Table 1) corresponding to conserved protein sequence domains of the *A. thaliana*, *Oryza sativa*, and *Zea mays CCD7* and *CCD8* orthologues. The *CCD7* and *CCD8* genomic loci were cloned using the GenomeWalker Universal Kit (Clontech, http://www.clontech.com) as specified by the manufacturer and using specific oligonucleotides (Table 1). The complete *CCD7* and *CCD8* coding sequences were PCR amplified from vascular tissue cDNA using specific oligonucleotides (Table 1) and High-Fidelity DNA Polymerase (NEB). DNA fragments were excised from agarose gels, isolated with the Promega Gel Extraction Kit and ligated into the pGEM-T vector (Promega, http://www.promega.com). Plasmids containing the inserts were sequenced using an automated DNA sequencer (ABI PRISM 3730xl, Perkin Elmer) from Macrogen Inc. (Seoul, Korea). Computer-aided sequence similarity searches were made with the BLAST suite of programs at the National Centre for Biotechnology Information (NCBI; http://www.ncbi.nlm.nih.gov) Motif searches were done using PROSITE (http://expasy.hcuge.ch/sprot/prosite.html), TMPRED (http://www.isrec.isb-sib.ch/sofware/sofware.html), SignalP (http://www.cbs.dtu.dk/services/SignalP) and PSORT II (http://psort.nibb.ac.jp).

**Table 1:** **Primer sequences used for *CsCCD7 *** **and *CsCCD8 *** **genes cloning and analysis**

	**Sequences**	**Primers (5′-3′)**
Degenerate primers	CCD8-F0	GTSGTGAGRATGGAASCHGG
	CCD8-F1	GNCAYYTNTTYGYGGNTAYGC
	CCD8-F2	CNCCNYTNTAYAARTTYARTGGCA
	CCD8-F3	ATMCCAYTKGAYGGRAGC
	CCD8-R0	CCATCATCYTCWTSGGTGC
	CCD8-R1	CCAYTRAAYTTRTANARNGGNGT
	CCD8-R2	TNGTNARNGTRTTNGGRAARCART
	CCD8-R3	CCARCADCCRTGVARGCC
	CCD7-F0	GACGAYCAYGGYTCCACSGTSCAC
	CCD7-F1	CTCGACGGCCAYGGYTACCT
	CCD7-R1	CYCKRTGCGTRAACCRCCACGA
Genome walker	CCD8-F1	GGTGTGGTTATSTCTGTGATAAGTG
	CCD8-F2	CCAACTTCGAGGAGTTGCACGA
	CCD8-R1	ATGCTCATTCGGGTCGAGTGCT
	CCD8-R2	CTTTGTCGCTCCCATCCAATGGT
	CCD8-R3	TTGCATGGCCTCACAGCTCCA
	CCD8-R4	CAACTCTCCTTTGTCGCTCCCATC
	CCD8-R5	TGCCGTTGATGAAGTGGAAGGTCAG
	CCD8-R6	TGTTGGCGTTGTCGCTGAGTGAC
	CCD8-R7	AGAAGTTGTCGGTCTTCGGGACT
	CCD8-R8	TACATGCTAGCCACAAGGGCTCCA
	CCD8-R9	TGGTCTGGCGTTCGGTAGGTGCT
	CCD7-F3	TCTCGGTGAACCCAAGCCAGCA
	CCD7-F4	GCAGACATCACCCATATATCTGCT
	CCD7-F5	TCTCGGTGAACCCAAGCCAGCA
	CCD7-R1	CAAGACAAAAAGGAATGGCA
	CCD7-R2	CAGTCGTGGATCATCAACTGGTCTG
	CCD7-R3	TGTCTCCATCTGTTGTCTGGTTG
	CCD7-R4	ATGATGGAGCTTATGTTGGGTCA
cDNA isolation	CCD7-F	ATGCACTCCATTTCTCACCGC
	CCD7-R	TAGTGATTTCTGCGAGACCA
	CCD8a-F	ATGGCAGACGTAGGGATACTGA
	CCD8b-F	CATGCAAACAAACTTAATAGCT
	CCD8-R	CTTGTCCTCTATTAAATTACTTC
QRT-PCR expression analysis	CCD7-F	ACCTCCCCGTCATCCAAT
	CCD7-R	ATGACGGTTTCGGTCTCG
	CCD8-F	GAAGGGGTCGATCGAGGT
	CCD8-R	CGTCGCCGTACTCGAACT

### Phylogenetic analysis

To construct the phylogenetic tree, the amino acid sequences were aligned using the BLOSUM62 matrix with the ClustalW (http://www.clustal.org) algorithm-based AlignX module from MEGA Version 5.0 (http://www.megasoftware.net/mega.html). The alignments were saved and executed by MEGA Version 5.0 to generate a Neighbour Joining Tree with bootstrapping (5000 replicates) analysis and handling gaps with pairwise deletion.

### Real-time quantitative RT-PCR (qPCR)

Total RNA was isolated from apical buds, secondary buds, vascular tissue from apical buds, vascular tissue from secondary buds, roots, stigmas and leaves by grinding the tissue in liquid nitrogen to a fine powder and extracting in 1 ml of Trizol reagent (Gibco-BRL) per 100 mg of tissue fresh weight, according to the protocol of the manufacturer. The RNA was resuspended in 100 μl of RNase-free water and treated with RQ1 RNase-free DNase (Promega). The quantitative RT-PCR was carried out on cDNA from 10 biological samples for each analysed tissue; reactions were set up in GoTaq^®^ qPCR Master Mix (Promega) according to manufacturer’s instructions, with gene-specific primers (0.125 μM) in a final volume of 25 μl. The primers were designed by using Primer3 program (http://frodo.wi.mit.edu/). Primer sequences are listed in Table 1. The constitutive expression gene 18SrRNA was used as a reference gene. The cycling parameters of qPCR consisted in an initial denaturation at 94°C for 5 min; 40 subsequent cycles of denaturation at 94°C for 20 s, annealing at 58°C for 20 s and extension at 72°C for 20 s; and finally extension at 72°C for 5 min. Assays were conducted with a StepOne™ Thermal Cycler (Applied Biosystems, California, USA) and analyzed using StepOne software v2.0 (Applied Biosystems, California, USA). Following reactions, DNA melt curves were created for each primer combination to confirm the presence of a single product.

### Statistical analysis

One-way analysis of variance (ANOVA) was performed on all data sets by using GenStat for Windows. When needed, data were also subjected to Student’s *t*-test.

## Abbreviations

ABA: Abscisic acid; CCD: Carotenoid cleavage dioxygenase; GAs: Gibberellins; IAA: Indole acetic acid; PCR: Polymerase chain reaction; SLs: Strigolactones; qPCR: Real-time quantitative RT-PCR.

## Competing interests

The authors declare no competing interests.

## Authors’ contributions

ARM carried out the physiology studies and drafted the manuscript. OA carried out the real-time PCR experiments and helped to draft the manuscript. RMCP and AGC carried out the determination of hormone levels. KY and JALR carried out the determination of strigolactones and helped to draft the manuscript. RVM participated in the design of the study, performed the statistical analysis and participated in the interpretation of data. LGG conceived of the study, participated in its design and coordination and helped to draft the manuscript. All authors read and approved the final manuscript.

## Supplementary Material

Additional file 1: Figure S1 Each sprouted axillary bud will form a new replacement corm. A) The developed new corms are formed from apical buds. B) The developed new corms are formed from axillaries buds, sprouted and developed after decapitation of the apical bud. Click here for file

Additional file 2: Figure S2 Hormone treatments induced different effects on sprouting in saffron corms. Signal + refers to the removal of the apical bud. Signal – refers to intact corms with their apical bud. GA3, gibberellic acid; NAA, 1-naphthalene acetic acid; BAP, benzylaminopurine. Surface sterilize corms were grew in MS medium containing or not different hormones at 100 μM final concentration. Picture was taken 10 days after treatment. The table shows the average length of the sprouted axyllary buds. Click here for file

Additional file 3: Figure S3 Homologues of the CCD8 gene in different plant species obtained by using CsCCD8a and b amino acid sequences in the Phytozome v9.1 data base. Synteny of each gene is shown as well as the exons distribution. Exon are shown in blue boxes and introns are shown as grey lines. Click here for file
