# Supplementary material for: Apical dominance in saffron and the involvement of the branching enzymes CCD7 and CCD8 in the control of bud sprouting
Source: BMC Plant Biol. 2014 Jun 19;14:171. doi: 10.1186/1471-2229-14-171 (PMC4077219; doi:10.1186/1471-2229-14-171)
Supplement: Additional file 1: Figure S1 — Each sprouted axillary bud will form a new replacement corm. A) The developed new corms are formed from apical buds. B) The developed new corms are formed from axillaries buds, sprouted and developed after decapitation of the apical bud. [file 1471-2229-14-171-S1.ppt]

## Slide 1
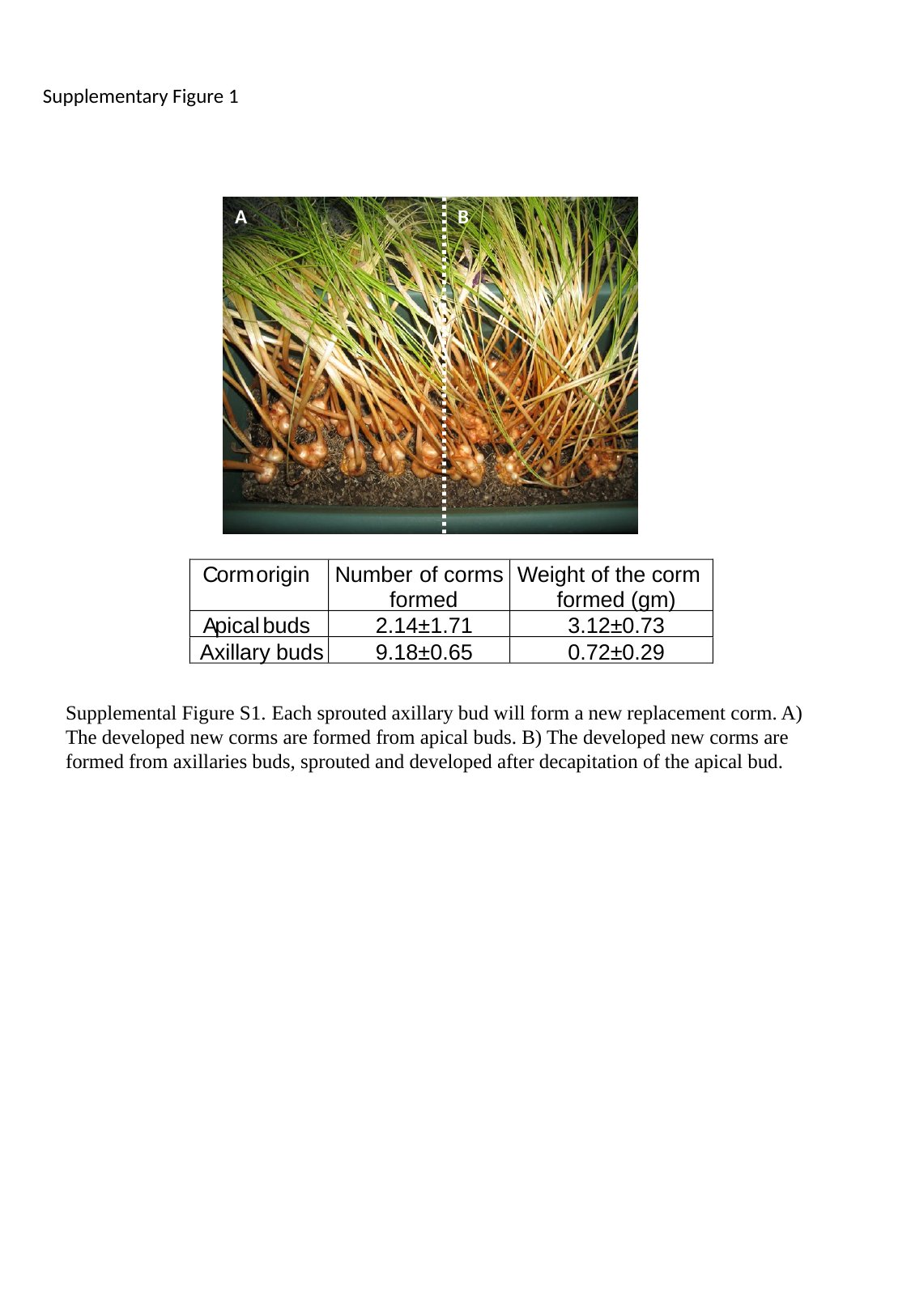

Supplementary Figure 1
A
B
C
orm
origin
Number
of corms
Weight
of the corm
formed
formed (gm)
A
pical
buds
2.14±1.71
3.12±0.73
Axillary buds
9.18±0.65
0.72±0.29
Supplemental Figure S1. Each sprouted axillary bud will form a new replacement corm. A) The developed new corms are formed from apical buds. B) The developed new corms are formed from axillaries buds, sprouted and developed after decapitation of the apical bud.
