# Supplementary material for: Apical dominance in saffron and the involvement of the branching enzymes CCD7 and CCD8 in the control of bud sprouting
Source: BMC Plant Biol. 2014 Jun 19;14:171. doi: 10.1186/1471-2229-14-171 (PMC4077219; doi:10.1186/1471-2229-14-171)
Supplement: Additional file 2: Figure S2 — Hormone treatments induced different effects on sprouting in saffron corms. Signal + refers to the removal of the apical bud. Signal – refers to intact corms with their apical bud. GA3, gibberellic acid; NAA, 1-naphthalene acetic acid; BAP, benzylaminopurine. Surface sterilize corms were grew in MS medium containing or not different hormones at 100 μM final concentration. Picture was taken 10 days after treatment. The table shows the average length of the sprouted axyllary buds. [file 1471-2229-14-171-S2.ppt]

## Slide 1
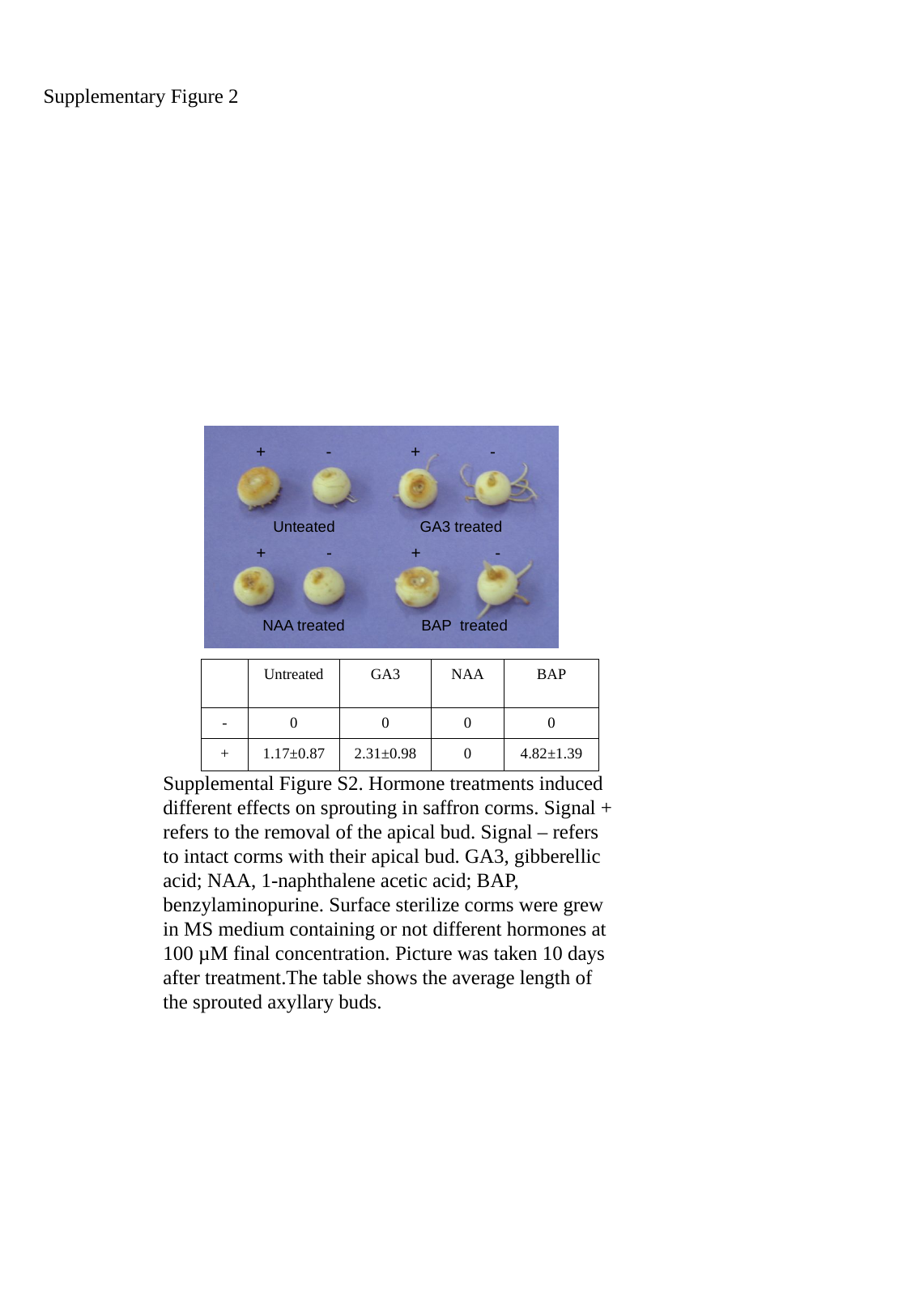

Supplementary Figure 2
+ - + -
+ - + -
Unteated GA3 treated
NAA treated BAP treated
| | Untreated | GA3 | NAA | BAP |
| --- | --- | --- | --- | --- |
| - | 0 | 0 | 0 | 0 |
| + | 1.17±0.87 | 2.31±0.98 | 0 | 4.82±1.39 |
Supplemental Figure S2. Hormone treatments induced different effects on sprouting in saffron corms. Signal + refers to the removal of the apical bud. Signal – refers to intact corms with their apical bud. GA3, gibberellic acid; NAA, 1-naphthalene acetic acid; BAP, benzylaminopurine. Surface sterilize corms were grew in MS medium containing or not different hormones at 100 µM final concentration. Picture was taken 10 days after treatment.The table shows the average length of the sprouted axyllary buds.
