# Supplementary material for: Apical dominance in saffron and the involvement of the branching enzymes CCD7 and CCD8 in the control of bud sprouting
Source: BMC Plant Biol. 2014 Jun 19;14:171. doi: 10.1186/1471-2229-14-171 (PMC4077219; doi:10.1186/1471-2229-14-171)
Supplement: Additional file 3: Figure S3 — Homologues of the CCD8 gene in different plant species obtained by using CsCCD8a and b amino acid sequences in the Phytozome v9.1 data base. Synteny of each gene is shown as well as the exons distribution. Exon are shown in blue boxes and introns are shown as grey lines. [file 1471-2229-14-171-S3.pptx]

## Slide 1
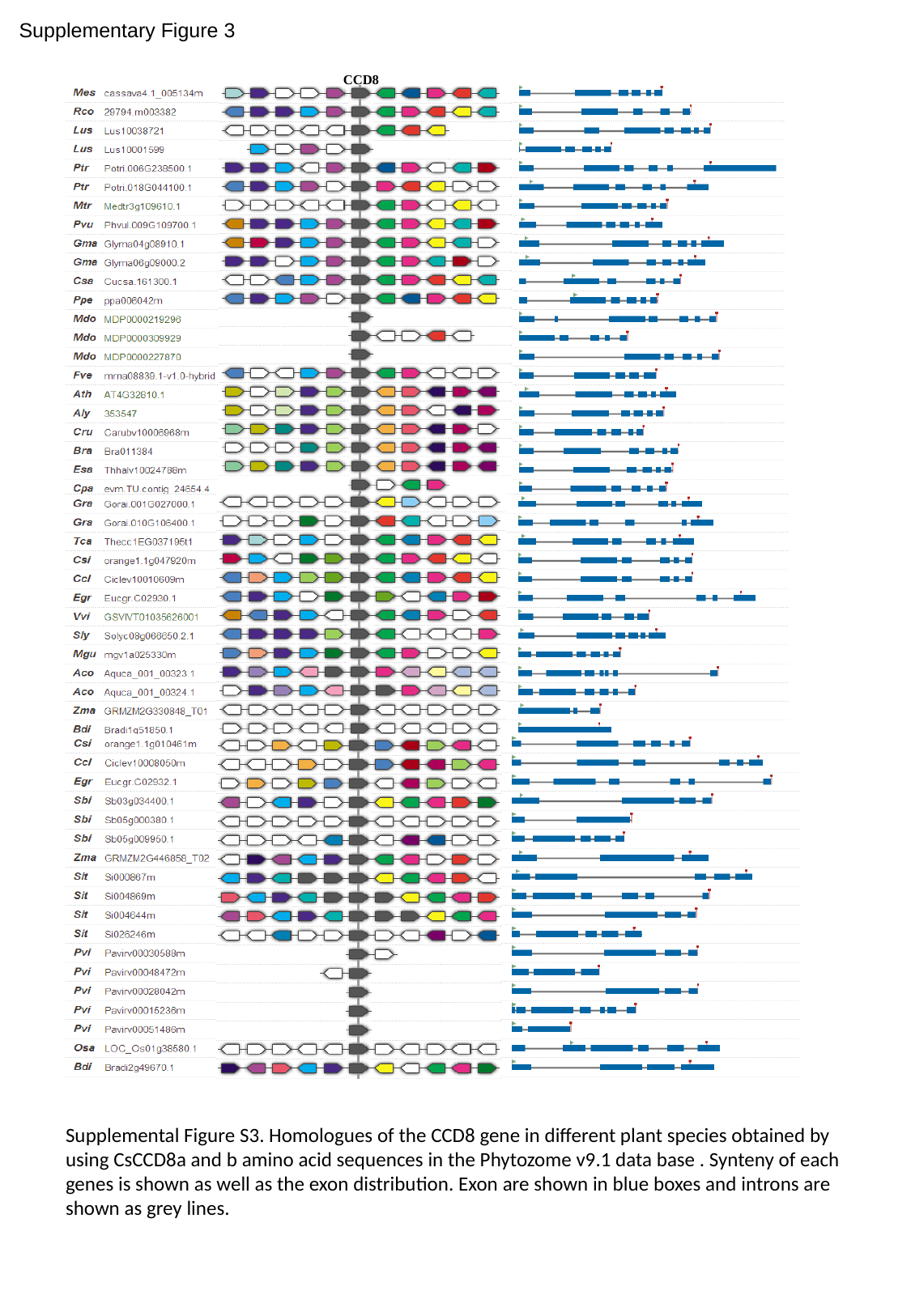

Supplementary Figure 3
CCD8
Supplemental Figure S3. Homologues of the CCD8 gene in different plant species obtained by using CsCCD8a and b amino acid sequences in the Phytozome v9.1 data base . Synteny of each genes is shown as well as the exon distribution. Exon are shown in blue boxes and introns are shown as grey lines.
